# Experience with Implementation of Pulse Oximetry Screening for Critical Congenital Heart Disease in a Low–Middle-Income Country

**DOI:** 10.3390/ijns12030062

**Published:** 2026-07-30

**Authors:** Abena Adaboh, Daem Celestin, Alex Agyekum, Nana Serwaa Osei, Araba Mensah, Sadath Sayeed, Nana-Akyaa Yao

**Affiliations:** 1 David Geffen School of Medicine, University of California, Los Angeles, CA 90095, USA; 2 Korle Bu Teaching Hospital, Accra GA-221-1570, Ghana

**Keywords:** critical congenital heart disease, implementation, pulse oximetry screening, challenges, sub-Saharan Africa, low resource

## Abstract

The adoption of pulse oximetry screening (POS) in low- and middle-income countries remains limited. We describe the development, implementation, and evaluation of a POS program at two of the leading tertiary centers in Accra, Ghana, based on a modified American Academy of Pediatrics protocol. Screening was conducted by trained research assistants. Operational experiences of research assistants were evaluated using a mixed-methods approach, including post-implementation surveys (*n* = 14) and focus group discussions (*n* = 10). Although participants felt comfortable with the level of instruction, they recommended incorporating simulation exercises to strengthen management of failed screens. Key challenges included difficulty locating newborns due to fragmented wards and paper-based records, equipment durability issues, and complex coordination for echocardiography following failed screens. Early discharge practices and high neonatal intensive care unit admissions reduced screening coverage among high-risk infants. Despite limited referral systems and surgical capacity, the POS pilot increased provider awareness, improved diagnostic clarity and demonstrated feasibility. Sustainable expansion will require more efficient referral networks as well as investment in pediatric cardiology training and cardiac surgical infrastructure.

## 1. Introduction

Pulse oximetry screening (POS) is a well-validated modality for detecting critical congenital heart diseases (CCHDs) [1,2]. POS is the standard of care in most developed healthcare systems, serving as an adjunct to the newborn physical exam and antenatal ultrasound for high CCHD detection rates [3,4,5,6]. In recent years, growing interest in POS in developing countries across Asia, Africa and the Middle East has produced multiple CCHD implementation pilot projects [7,8,9,10,11,12,13]. Unfortunately, progress in these regions is hindered by broad resource limitations and country-specific challenges [14,15]. A deeper understanding of these unique contextual factors is vital to successful, sustainable POS implementation.

Ghana, a lower–middle-income country with an estimated population of 31 million and approximately 800,000 according to 2023–2024 national census data, faces substantial challenges in the early detection of congenital heart diseases (CHDs) [16]. A retrospective EHR study from a tertiary center in the country estimated a CHD incidence of roughly 0.8% of live births, although case identification was limited by incomplete documentation and reliance on paper records [17]. Like many sub-Saharan African countries, Ghana still lacks a standardized screening protocol for CCHDs. Most newborns are discharged without a physician-performed examination. The 2013 Strategy and Action Plan from Ghana’s Ministry of Health recommends at least 24 h of postnatal observation with immediate assessment at birth and a systematic head-to-toe examination within the first hour and again prior to discharge in accordance with World Health Organization guidelines [18]. However, coverage remains low: an estimated 23% of newborns receive an examination within 48 h of birth and 30% within the first week [19]. In practice, postnatal care typically consists of routine checks limited to vital signs and basic anthropometric measurements.

Previous efforts to evaluate congenital heart disease in the country have largely been restricted to evaluating the utility of the cardiovascular physical exam at birth or conducting retrospective analyses of electronic health records [20,21]. In response to these gaps, a study led by the country’s only pediatric cardiologist at the time was launched in 2024 to assess the feasibility and effectiveness of POS for CCHDs and to identify barriers to its widespread implementation. This paper describes the screening protocol’s development, reports implementation challenges, and discusses how such factors may impact success in similar resource-constrained contexts. A separate manuscript describes the screening results of nearly 6000 neonates from two high-volume tertiary hospitals in Accra [21].

## 2. Protocol Development

The development of the final study protocol was an iterative process that required adaptation to the realities of the Ghanaian healthcare setting. Informal discussions were held with hospital leadership, including the heads of the pediatric and obstetric departments, before and during the institutional review board approval process, followed by formal presentations to pediatric staff at both hospitals prior to study initiation. Strong concerns were raised regarding the potential burden that screening activities could place on already overstretched nursing personnel. To minimize disruption to clinical workflows and ensure standardized implementation while still allowing for interest building, dedicated research assistants were employed to conduct screening activities.

The original 2011 AAP-endorsed algorithm was modified to allow screening at ≤48 h given the routine practice of discharging healthy newborns before 24 h of life [22]. Although the initial screening process included a newborn exam by a pediatrician or pediatric resident alongside pulse oximetry, the exam component was discontinued at KBTH due to limited provider availability. To mitigate the potential for technological failures and minimize data entry errors, all data were first collected on paper and later entered into Redcap by dedicated data entry personnel.

## 3. Methods

### 3.1. Study Context

The POS study took place at Korle Bu Teaching Hospital (KBTH) and 37 Military Hospital (37MH). KBTH is the largest tertiary hospital in Ghana and the leading public referral center, serving patients across a wide socioeconomic spectrum with a delivery rate of approximately 6000 to 10,000 annual births. 37MH is the largest military tertiary health institution in the country, providing care for service personnel and the public with about 2500 to 4000 annual deliveries.

### 3.2. Study Participants

The study sample included research assistants who had participated in data collection for at least two months and were likely to have experienced daily fluctuations in workload.

### 3.3. Study Design

A mixed-methods approach was used to elicit the perspectives of research assistants involved in the pulse oximetry screening program. Quantitative data were collected using a structured Qualtrics questionnaire administered following study completion between 3 February and 31 March 2025, while qualitative data were obtained through focus group discussions (see Supplementary Materials). As no validated instruments specific to newborn pulse oximetry screening implementation in this setting were identified, the questionnaire and focus group guide were developed by the study team based on prior implementation experience, the relevant published literature, and informal discussions with research assistants and hospital staff. The instruments were designed to assess experiences across all stages of the screening process, including newborn identification, consent, data collection, pulse oximetry screening, referral coordination, and training. Questions were iteratively reviewed by the investigators to ensure clarity, minimize ambiguity, and establish face validity. Formal pilot testing was not conducted because of the limited number of research assistants available for participation.

Research assistants who elected to participate in the qualitative component were divided into two groups for 90 min focus group discussions conducted on 16 February and 23 February 2025. Discussions were facilitated by a lead co-investigator involved in the design and implementation of the screening program using a semi-structured discussion guide. Participants provided informed consent and permission for audio recording and transcription. The combination of structured survey responses and focus group discussions allowed for both quantitative assessment and in-depth exploration of research assistants’ experiences with program implementation.

### 3.4. Data Analysis

Survey data were analyzed using simple descriptive statistics [23]. Transcribed interviews were reviewed by the study team and general themes were documented. Inductive thematic analysis was used to generate coding topics and sub-topics. Finished codes were then used to evaluate the interviews using MAXQDA 24 software.

## 4. Results

Among the 18 research assistants who participated in the study and met inclusion criteria, 14 responded to the survey, and of those, 10 elected to participate in the focus group discussions. All research assistants who participated in the focus group discussions had dealt with at least one pulse oximetry failure. Data from both the survey and interviews were integrated to provide a more comprehensive understanding of the study experience.

### 4.1. Characteristics of Respondents

All survey respondents were between the ages of 20 and 39 and had at least a bachelor’s degree. The majority, 8/14 or 57%, were in the health field, with six newly graduated medical doctors (yet to begin post-education field training) and two medical students. While the other six had academic backgrounds outside of medicine—such as public health, psychology, computer science and business—5/6 had prior experience working as research assistants on multiple research projects at KBTH. All spoke English and at least one other language, most commonly Akan (85.7%). Nine were involved in screening at KBTH and five were based at 37MH.

### 4.2. Training

Participants described having an average of 2 days of 1–2 h long hands-on practice sessions, beginning with a detailed review of the study protocol followed by pulse oximetry practice under the instruction of members of the study team or experienced research assistants. The majority (71.4%) reported that this was sufficient for consenting parents and performing pulse oximetry, while 64.3% felt it was adequate for their understanding and comfortability using the study algorithm (Table 1). Approximately one-third of participants expressed that additional in-person training would have been helpful. During the discussions, several elaborated that they felt unprepared for the logistical complexities of managing failed screening cases (discussed further under “reporting fails”). One participant recommended incorporating a simulated failed case scenario into future training sessions to better equip healthcare workers.

### 4.3. Screening Process (Figure 1)

*Locating newborns:* All participants identified locating newborns as the most time-consuming task in their workflow. Patient tracking was noted to be considerably more difficult at KBTH compared to 37MH. As elaborated by participants, the maternity unit spanned six separate floors, each functioning semi-independently with discrete staff nurses and paper-based record-keeping. There were similarly two separate labor and recovery wards. Participants described a complex and time-consuming process involving the review of multiple logbooks, direct inquiries with ward nurses, informal use of the electronic health system (which was frequently not up to date) and searching bed to bed to locate mothers/newborns. Reported search times at KBTH ranged from 5 min to an hour, depending on delivery volume. In contrast, 37MH—with fewer, clustered maternity wards on a single floor and lower delivery rates—allowed for quicker identification, typically within five minutes.

**Figure 1:**
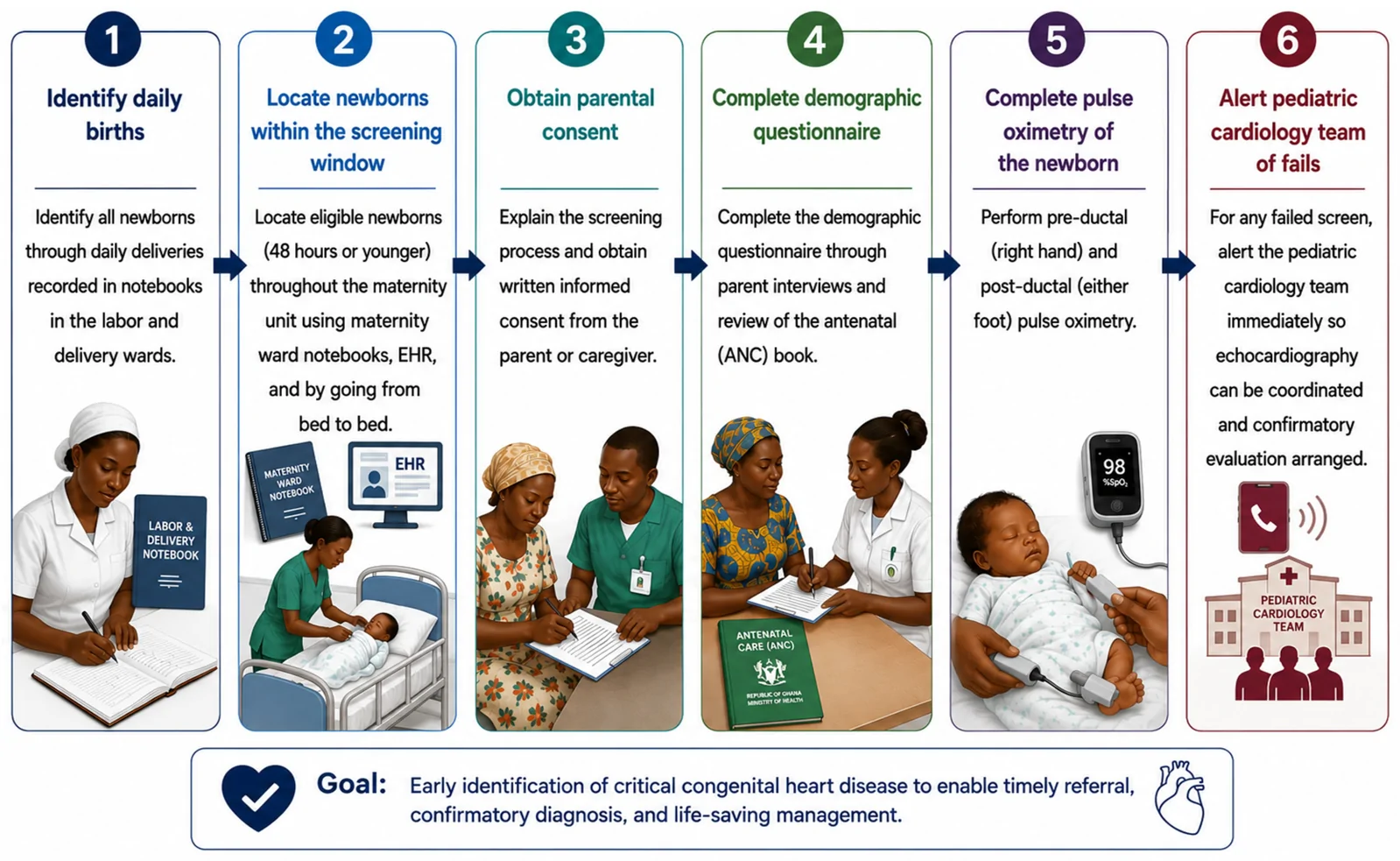
Critical congenital heart disease (CCHD) screening process.

*Consenting*: Participants consistently described obtaining parental consent for newborn screening as a relatively straightforward process, typically taking anywhere between 1 and 2 min. However, a few issues were noted. There were often language barriers with mothers who did not speak English, Akan, or Ga, the three most widely spoken languages amongst the staff and patients. Additional obstacles stemmed from parental fears that the screening process might harm the newborn, anxiety about the possibility of detecting a heart condition, and occasional expectations of financial incentives. Approximately 86% (12 out of 14) of research assistants agreed that future consent processes could be improved by providing prenatal and antenatal education on screening, as well as visual aids such as posters or diagrams illustrating the procedure.

*Demographic questionnaire*: Similar to the process of consenting, administering the demographic questionnaire was felt to be uncomplicated. All research assistants used the antenatal book to supplement the maternal interview, as it contained detailed prenatal and antenatal medical information. Given that many mothers were unfamiliar with medication names and formal diagnoses, research assistants relied on the antenatal book to clarify any discrepancies between self-reported and documented histories. Duration of administering the questionnaire ranged from 2 to 4 min and was highly dependent on how complete and readily accessible the antenatal book was.

*Pulse oximetry*: Table 2 summarizes participants’ experiences with pulse oximetry. Masimo RAD-97 pulse oximeters (Masimo Corporation, Irvine, California, USA) with multisite Y-I reusable sensors and soft foam wraps, suitable for neonates, were used. Although interview responses reflected a high level of comfort with operating the pulse oximeter, most participants reported recurring technological difficulties emerging around the fourth month of the study. Most noted issues with the reusable Velcro strap and the sensor as their biggest challenge (Table 2). In addition to equipment-related challenges, non-technological factors—such as infant crying (85.7%) and fussing (71.4%)—were commonly identified as key contributors to delayed pulse oximetry readings, defined as taking one minute or more per limb.

*Reporting failures*: Survey findings pertaining to the process of reporting failures are summarized in Table 3. Research assistants described relative ease in following the study algorithm but described difficulties communicating effectively with the study team, clinical staff and parents while coordinating transport to an echocardiography (echo) lab. KBTH had an echo lab located in a different building from the maternity complex; however, 37MH had no pediatric echo facilities, requiring babies to be transported to KBTH or a nearby pediatric cardiology clinic for echocardiographic diagnosis. One research assistant reported that “everything happens in a flash,” emphasizing the importance of communication to help facilitate the next steps. Others also noted consistent issues with long wait times once the mother and baby were brought to an echo lab (due to long pediatric cardiology patient lists), causing considerable discomfort for both the newly delivered mother and infant.

## 5. Discussion

Our findings build on previous reported LMIC experiences with incorporating CCHD screening into routine clinical practice [7,8,9,10,11,12]. It is important to acknowledge that the perspectives we captured did not include the viewpoints of key clinical staff such as nurses who would ultimately perform pulse oximetry screening as part of routine care or physicians responsible for clinical oversight. Consequently, our findings may not fully reflect the barriers and facilitators encountered during long-term integration into clinical practice.

Nevertheless, the screening procedures and implementation recommendations developed through this project may help address many of the operational challenges associated with broader adoption. In particular, given the well-documented knowledge gaps surrounding CCHDs and POS among providers in low- and middle-income countries, as well as the need for ongoing education to maintain implementation fidelity, many of the training needs identified by research assistants are likely to be relevant to frontline nursing staff [8,24,25]. Below, we incorporate our own informal experiences as study investigators with the reported findings and highlight a few broad themes for discussion.

### 5.1. Challenges in Maintaining Training Fidelity

Effective, standardized training has been shown to support successful screening implementation by enhancing provider knowledge, increasing screening coverage, and reducing error [26,27]. At study initiation, training and education sessions were provided to research assistants and clinical providers within the pediatric department. While most research assistants expressed confidence in applying the CCHD algorithm, some noted that comprehension and retention could be improved with the incorporation of videos, diagrams and simulations in addition to hands-on training sessions—indicating the potential value of a multimodal training approach. A key barrier to quality assurance was the inability to sustain ongoing training workshops for research assistants and to regularly orient newly rotating physician trainees to the study protocol. This was primarily due to limited availability of both clinical and study personnel, as well as diminishing engagement over time. As a result, a recurring issue was a lack of awareness amongst some clinical providers, occasionally delaying timely care for affected newborns. Similar training challenges in other low-resource settings have been linked to protocol deviations and suboptimal communication with families regarding screening [10,12,28].

### 5.2. Operational Barriers to Effective Pulse Oximetry Screening

The screening process was divided into pre-screening documentation, which included consenting, followed by pulse oximetry measurement. Delays to pre-screening often came in the form of extended counseling for parents due to language barriers, limited literacy and anxiety. These setbacks are manageable with dedicated research assistants but disruptive to clinical care when nursing staff are expected to perform such duties. In a South African study, nurses cited the time burden of obtaining consent and completing documentation amidst heavy workloads as a major contributor to reduced screening rates [10]. In theory, as screening moves to a standard clinical practice (like obtaining birth weight and vital sign measurements) rather than an investigation into its implementation feasibility, the requirement and associated time for consenting parents could be reduced. However, adding another clinical task to already heavily burdened and often short-staffed nurses requires a structured and systematic approach, buy-in from all clinical stakeholders, and well-demarcated lines of responsibility and accountability.

### 5.3. Challenges Related to Record-Keeping and Patient Tracking

Incorporation of electronic health records (EHRs) into screening workflows has been shown to enhance the quality and effectiveness of screening programs [4]. For example, in Saudi Arabia, universal integration of the screening registry, national birth notification system and screening protocol has resulted in compliance rates exceeding 98% [13,29]. In sharp contrast, the absence of a reliable, centralized, EHR system in our setting significantly hindered basic logistical tasks such as compiling a list of daily deliveries and locating newborns and their mothers, particularly at KBTH. This challenge, alongside early discharges and NICU admissions, was a key contributor to reduced screening rates. Additionally, reliance on paper-based antenatal records, which were at times incomplete, inconsistently stored, or misplaced, further disrupted documentation and workflow efficiency.

### 5.4. Challenges in Post-Screening Evaluation and Access to Definitive Care

Ideally, a failed screen should trigger prompt clinical assessment and evaluation including echocardiography to identify causes of hypoxemia [30]. In our study, the lack of integration between departments required research assistants to manually notify and coordinate the activities of the primary pediatric team, cardiology research personnel, and obstetric staff—a cumbersome and time-consuming process. Limited availability of echocardiographers and echo facilities necessitated transporting newborns to different buildings or external centers, resulting in delays of several hours, similar to challenges reported in Morocco [9]. Even when CCHDs were diagnosed, in-country treatment options were limited to pharmacologic or palliative care, with surgical interventions only possible through costly and time-intensive referrals abroad.

### 5.5. Early Discharges and Barriers to Post-Discharge Follow-Up

The reality of early discharges for uncomplicated deliveries in low-resource settings has been well documented, necessitating modified protocols that allow for screening in <24 h despite the risk of higher false positive rates [7,8,11,28]. In our study, 74% of infants were screened within the first 24 h of life with an overall false positive rate of 0.14% compared to rates as low as 0.05% reported in studies screening at ≥24 h [1].

Post-discharge follow-up of newborns who failed screening was difficult to coordinate due to the heavy clinical workload of the pediatric cardiology team. This limited the ability to establish a standardized follow-up schedule and closely monitor clinical progress. Furthermore, assessing false negative rates, essential for evaluating screening effectiveness, was not possible due to the absence of centralized surveillance systems to track newborns who passed or missed screening [4,31].

### 5.6. Barriers in Ensuring Equitable Screening for High-Risk Newborns

**Figure 2:**
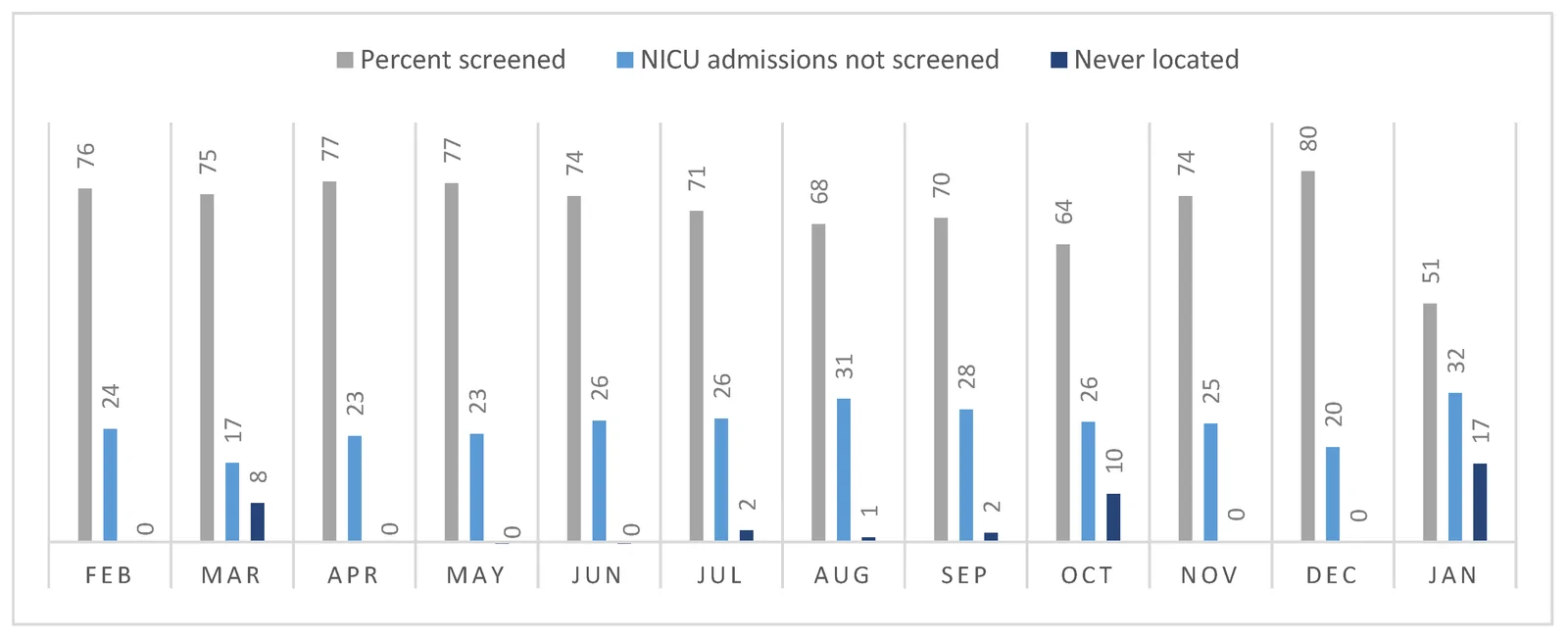
Screening rate at Korle-Bu Teaching Hospital.

While POS is traditionally targeted at asymptomatic newborns, recent studies from the United States have demonstrated the feasibility and potential benefits of systematic screening in the NICU despite a higher false positive rate from prevalent lung pathology and associated oxygen requirements [1,4,32,33]. Nonetheless, most studies in low-resource regions continue to exclude NICU populations, in line with prevailing guidelines such as those from the AAP [4,8,9,10,28,34]. In our setting, where diagnostic limitations exist across hospital units, we aimed to screen all newborns regardless of clinical status, provided they were not on oxygen or had been weaned off. However, this proved challenging, as symptomatic newborns were typically admitted directly from the labor wards to the NICU, started on oxygen, and often lost to follow-up due to inter-unit transitions, death or aging beyond the screening window while still on oxygen. This issue was most pronounced at KBTH. As shown in Figure 2, approximately 20% of total deliveries were admitted to the NICU at KBTH. Most of the NICU admissions were not screened. This screening gap was particularly concerning, as it disproportionately affected the highest-risk newborns who stood to benefit the most from early detection of critical congenital heart defects.

### 5.7. Ethical Considerations and Benefits of Screening

The ethics of CCHD screening in low-income settings are complex. Its utility is reasonably questioned in contexts where definitive intervention is largely unavailable and concerns regarding cost-effectiveness, sustainability, parental distress, and additional strain on already overburdened health systems are substantial. However, the impact of screening programs should not be evaluated solely on the basis of mortality reduction. In Ghana—where only a single pediatric cardiologist served the country at the time of study initiation—screening increased awareness of congenital heart disease among families and healthcare providers, provided trainees with rare exposure to pediatric cardiology, and improved early recognition and diagnostic clarity. Even in the absence of surgical access, identification enabled clinical stabilization, anticipatory counseling, palliative planning, informed discharge preparation for less severe lesions, and, in select cases, referral for care abroad.

**Figure 3:**
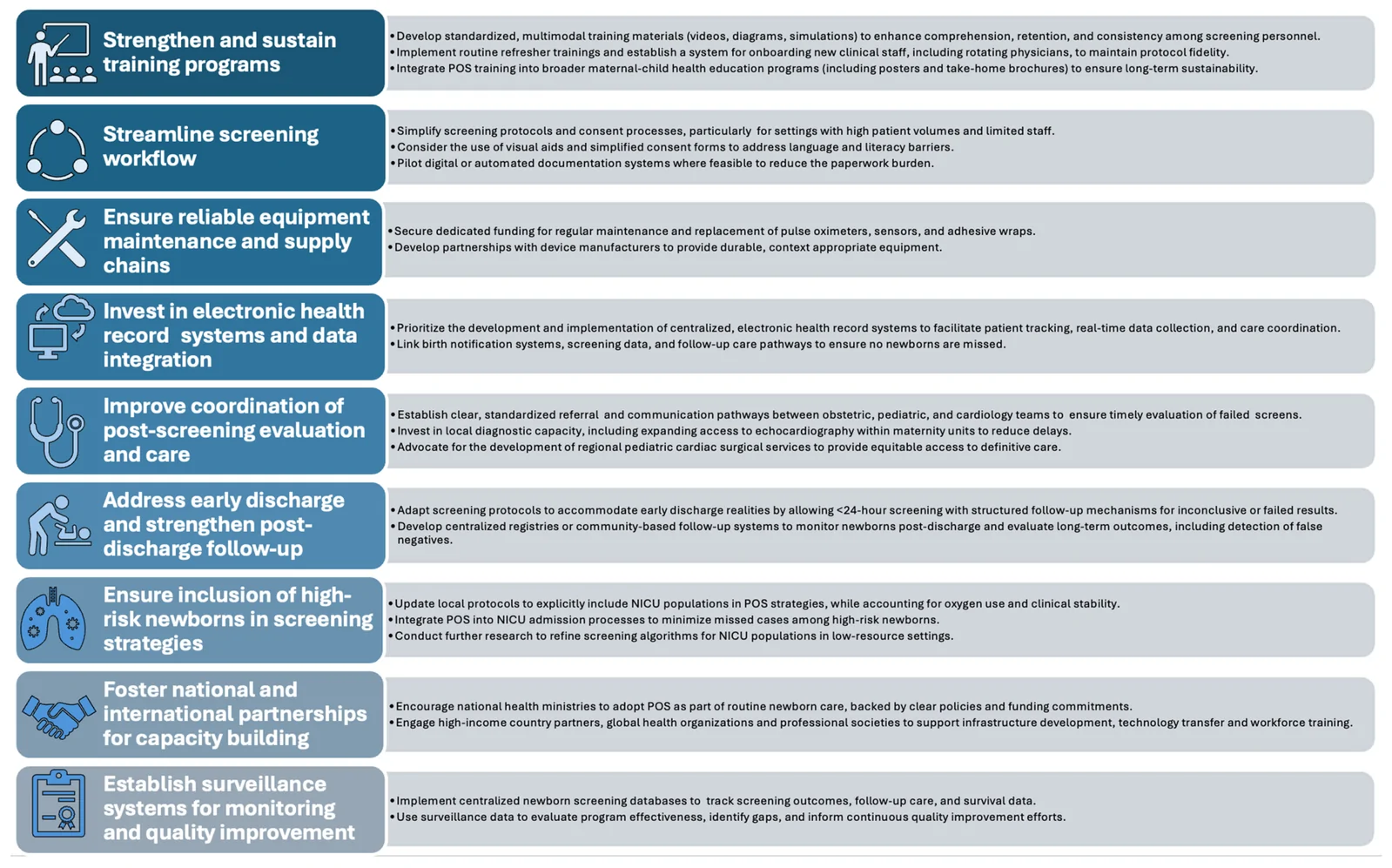
Recommendations to strengthen newborn pulse oximetry screening in low-resource settings.

Beyond the detection of CCHD, POS has increasingly been recognized as a valuable tool for identifying other clinically significant hypoxemic conditions, including neonatal sepsis, pneumonia, persistent pulmonary hypertension of the newborn, and primary respiratory disorders [8,35]. Consistent with these observations, two newborns with clinically important non-CHD conditions were identified through screening [21]. Moreover, for families, early diagnosis also provides diagnostic certainty in a socio-cultural context where neonatal deaths may otherwise be attributed to spiritual or supernatural causes and reduces expenditures from repeated healthcare encounters and diagnostic odysseys for infants with undiagnosed cyanotic heart disease. As a pilot initiative, this program demonstrated the feasibility and laid groundwork for integrating other low-cost noninvasive neonatal screening strategies. Drawing on these experiences, we propose several practical recommendations to guide future implementation efforts and scale-up initiatives (Figure 3).

## 6. Study Limitations

This study has important limitations. First, the relatively small number of research assistants involved in screening resulted in a limited sample size for both the interviews and survey, restricting the breadth of perspectives obtained. Second, our findings were derived from two tertiary referral centers and may not be generalizable to lower-level healthcare facilities. At present, tertiary centers are best positioned to support pulse oximetry screening programs because they possess the infrastructure, personnel, and diagnostic capacity required for timely confirmatory echocardiography and clinical management of infants with abnormal screening results. As such, implementation in non-tertiary settings may face additional challenges not captured in this study. Finally, by focusing primarily on the experiences of research assistants, the interviews and surveys emphasized the operational aspects of screening implementation rather than broader health system and structural barriers. We sought to contextualize these wider challenges within the discussion.

## 7. Conclusions

Our findings highlight the need to address national and hospital-specific healthcare barriers to universal pulse oximetry screening in low- and middle-income countries. We urge governments in LMICs to focus policy efforts and resources on incorporating screening into routine care. We also call on high-income countries to support this endeavor by investing in capacity building. Immediate group action and shared responsibility are needed to reduce global disparities in the diagnosis and treatment of congenital heart disease.

## Figures and Tables

**Table 1:** Training.

ITEM (Select All That Apply)	Training Was Sufficient	More Hands-On/In-Person	Video Demonstration	Diagrams or Figures	Written Instructions
How could your pulse oximetry training have been improved?	10 (71.4%)	5 (35.7%)	2 (14.3%)	2 (14.3%)	0 (0.0%)
How could your training on consenting parents have been improved?	10 (71.4%)	3 (21.4%)	3 (21.4%)	1 (7.1%)	0 (0.0%)
How could your understanding and use of the study algorithm have been improved?	9 (64.3%)	4 (28.6%)	1 (7.1%)	1 (7.1%)	1 (7.1%)

**Table 2:** Pulse oximetry.

Item	N (Percent)
The designated pulse oximeter was accessible or easy to find	
Strongly agree	10 (71.4%)
Somewhat agree	1 (7.1%)
Neither agree nor disagree	1 (7.1%)
Somewhat disagree	1 (7.1%)
Strongly disagree	1 (7.1%)
What was the primary issue you had using the pulse oximeter?	
Issues with sensor strap	8 (57.1%)
Issues with sensor	5 (35.7%)
Low battery	1 (7.1%)
How long did it take to obtain a pulse oximetry reading on average?	
<1 min	7 (50.0%)
1–2 min	6 (42.9%)
2–3 min	1 (7.1%)
What were obstacles to obtaining a pulse oximetry reading in <1 min apart from technological problems (select all that apply)	
Crying baby	12 (85.7%)
Restless/fussy baby	10 (71.4%)
Interruption from parent	5 (35.7%)
Unable to physically access right hand or foot	5 (35.7%)
Interruption from nursing staff	2 (14.3%)

**Table 3:** Reporting and managing failed cases.

Item	N (Percent)
I was able to follow the study algorithm relatively easily once a failed baby was identified	
Strongly agree	7 (53.8)
Somewhat agree	5 (38.5)
Neither agree nor disagree	0 (0.0)
Somewhat disagree	1 (7.7)
Strongly disagree	0 (0.0)
What was most challenging about the study protocol? Select all that apply.	
Nothing was confusing	7 (53.8)
Getting in touch with a member of the study team to plan an echo	7 (53.8)
Identifying absolute vs. relative fails	2 (15.4)
Timing of repeat checks	1 (7.7)
In what ways might you have deviated from the study protocol? Select all that apply.	
Repeating pulse oximetry on absolute fails (saturations less than 90)	6 (50.0)
Screening babies > 48 h old	3 (25.0)
Not repeating pulse oximetry on infants with >3 saturation difference between R hand and foot	3 (25.0)
Who did you contact for a failed baby? Select all that apply.	
Call/text appointed study team point of contact	12 (92.3)
Report in research assistant group chat	9 (69.2)
Call/text study PI	7 (53.8)
Let a member of the hospital clinical team know	5 (38.5)
How long did you typically take to contact a member of the study team for a failed baby?	
Immediately	8 (61.5)
Within 30 min	4 (30.8)
Within 2 h	1 (7.7)

## Data Availability

All relevant data are included in the article or uploaded as Supplementary Materials.

## Supplementary Materials

The following supporting information can be downloaded at https://www.mdpi.com/article/10.3390/ijns12030062/s1: focus group discussion guide, research assistant feedback survey, and focus group discussion consent form.

## Abbreviations

POS	Pulse oximetry screening
CCHD(s)	Critical congenital heart disease(s)
AAP	American Academy of Pediatrics
KBTH	Korle-Bu Teaching Hospital
37MH	37 Military Hospital
HER	Electronic health record
NICU	Neonatal intensive care unit
LMIC(s)	Low–Middle-Income Countries
