# Supplementary material for: Experience with Implementation of Pulse Oximetry Screening for Critical Congenital Heart Disease in a Low–Middle-Income Country
Source: Int J Neonatal Screen. 2026 Jul 30;12(3):62. doi: 10.3390/ijns12030062 (PMC13511312; doi:10.3390/ijns12030062)
Supplement: Supplementary file 1 [file IJNS-12-00062-s001.zip › IJNS-4203110-supplementary/Supplemental files/Focus group discussion consent form.pdf]

FOCUS GROUP DISCUSSION CONSENT FORM

<u>Purpose</u>
The critical congenital heart disease (CCHD) screening research team, led by Professor Nana Akyaa Yao, has invited you to participate in a focus group. The group aims to understand the experiences and perceived challenges of research assistants from training to study completion. The information gleaned from this focus group will be used to improve screening practices in hospitals across the country.

<u>Procedure</u>
As part of this study, you will be placed in a group of 4-5 individuals. A moderator will ask you several questions while facilitating the discussion. As approved through the Korle Bu Teaching Hospital and 37 Military Hospital IRB, this focus group will be audio-recorded, and a note-taker will be present. However, your responses will remain confidential, and no names will be included in the final report.

You can choose whether to participate in the focus group, and you may stop at any time throughout the study.

Please note that there are no right or wrong answers to focus group questions. The CCHD screening research team wants to hear varying viewpoints and wants everyone to contribute their thoughts. Out of respect, please refrain from interrupting others. However, feel free to be honest even when your responses counter those of other group members.

<u>Benefits and Risks</u>
You will receive compensation for your participation; however, more broadly, your involvement will benefit newborns across Ghana by improving the detection of critical congenital heart disease. No risks are anticipated beyond those experienced during an average conversation.

<u>Confidentiality</u>
Should you choose to participate, you will be asked to respect the privacy of other focus group members by not disclosing any content discussed during the study. The CCHD study team members will analyze the data, but—as stated above—your responses will remain confidential, and no names will be included in any reports.

<u>Contact</u>
If you have any questions or concerns regarding this study, please contact:

Professor Nana Akyaa Yao, *Principal Investigator*
Phone: +233202624002
Email: nanayao@doctors.org.uk

I understand this information and agree to participate fully under the abovementioned conditions.

____   _____/_____/__________

Name and Signature of Research Assistant   Date
