# Supplementary material for: Experience with Implementation of Pulse Oximetry Screening for Critical Congenital Heart Disease in a Low–Middle-Income Country
Source: Int J Neonatal Screen. 2026 Jul 30;12(3):62. doi: 10.3390/ijns12030062 (PMC13511312; doi:10.3390/ijns12030062)
Supplement: Supplementary file 1 [file IJNS-12-00062-s001.zip › IJNS-4203110-supplementary/Supplemental files/Focus group discussion guide.pdf]

RESEARCH ASSISTANT FOCUS GROUP INTERVIEW GUIDE

Questions and Probes	Themes
1. **Please describe your daily screening routine/a typical day** (*Allow them to come up with an exhaustive list of activities/tasks in chronological order*)<br><br>▪ Important responsibilities of a research assistant are to compile a list of births, consent parents, administer the screening questionnaire and perform pulse oximetry. If you were to rank these four in order of difficulty, which one would you rank first, second, third and fourth? Why?<br>▪ Specifically, which elements are challenging about the highlighted area?	"Icebreaker question"<br>**Tasks of a research assistant – Role Listing**
2. **How much time does it take to screen a baby on average?**<br>▪ How much time would you estimate is spent locating an eligible newborn? Consenting? Administering the screening questionnaire? Performing pulse oximetry?<br>▪ What factors delay each step of the process/what step takes the longest time?<br>▪ Do you think the most important activities to screening take up the most amount of time or is that not always true? Why?<br>▪ Are there instances when you are unable to complete screening due to time limitations?	**Time spent on Tasks**
3. **Did you feel your training was adequate?**<br>▪ How many hours/days of training/orientation did you receive on average?<br>▪ Could you describe the training process?<br>▪ Did you find instructions clear?<br>▪ What tasks were most challenging to grasp/master?<br>▪ Did you feel supported by the research admin team when challenges arose, or you had questions?<br>▪ What aspects of your training were most helpful? What would you change?<br>▪ What types of support did you routinely require over the course of the study?	**Training**
4. **Interactions with nursing staff/physicians/techs**<br>▪ How would you describe your typical interactions with nursing staff/physicians/techs?<br>▪ Of the medical staff, who did you interact with the most? Why?	**Interactions with hospital staff**

▪ Did you routinely need support from medical staff? For what tasks?<br>▪ What challenges did you face in your interactions? How did the challenges impact screening?<br>▪ What would improve your interactions?	
**5. Describe your process of reporting fails.** (Ask them to describe line of communication)<br>▪ Did you know who to reach out to on the research team? Who?<br>▪ Did you know who to reach out to on the clinical team? Who?<br>▪ Did you feel like everyone who needed to be made aware knew when a baby had failed pulse oximetry screening?<br>▪ How were parents informed of fails?<br>▪ How else did you facilitate the process of a failed baby obtaining an echocardiogram?	**Reporting fails**
**6. Overall, how do you feel about your role?**<br>▪ What is the one thing that you enjoyed or appreciated?<br>▪ What is the one thing that you disliked/detested about your role?<br>▪ If any research assistants quit along the way, what reasons did they give?<br>▪ What is the one thing that you would like to change?<br>▪ Is there any compelling story/impact case that you can share that encapsulates the value of the work that you did?	**Improvement Plan & Wrap Up**
