# Supplementary material for: Experience with Implementation of Pulse Oximetry Screening for Critical Congenital Heart Disease in a Low–Middle-Income Country
Source: Int J Neonatal Screen. 2026 Jul 30;12(3):62. doi: 10.3390/ijns12030062 (PMC13511312; doi:10.3390/ijns12030062)
Supplement: Supplementary file 1 [file IJNS-12-00062-s001.zip › IJNS-4203110-supplementary/Supplemental files/Research assistant feedback survey.pdf]

RESEARCH ASSISTANT FEEDBACK SURVEY

SECTION A: INTRODUCTION

You are invited to participate in this survey regarding the experiences of research assistants in the screening of newborns of newborns for critical congenital heart diseases at Korle-Bu Teaching Hospital and 37 Military Hospital from February 2024 to January 2025. This online survey will take approximately 5-10 minutes to complete. Participation is voluntary, and all responses will remain anonymous. You may skip any question you prefer not to answer. By submitting the survey, you indicate your consent to participate in this study.

If you have questions, please contact the study's principal investigator, Professor Nana-Akyaa Yao at nanayao@doctors.org.uk.

SECTION B: DEMOGRAPHICS

1. Age:
    a. 20 – 29
    b. 30 – 39
    c. 40 – 49
    d. 50 – 59
    e. 60 – 69

2. Highest educational attainment:
    a. Secondary school certificate
    b. Technical certificate
    c. Some college but did not graduate
    d. Bachelor's degree
    e. Graduate school
    f. Professional: nursing
    g. Professional: medicine

3. Area of educational specialty:
    a. Business
    b. Communication
    c. Computer Science
    d. Economics
    e. Education
    f. Engineering
    g. Medicine
    h. Nursing
    i. Psychology
    j. Public Health

    k. Other:

4. Languages spoken (select all that apply):
    a. English
    b. Akan
    c. Ga/Adangbe
    d. Ewe
    e. Hausa
    f. French
    g. Other:

5. How long were involved in the study?
    a. <1 month
    b. 1-2 months
    c. 3-4 months
    d. 5-6 months
    e. 6+ months

6. Primary hospital site:
    a. Korle-Bu Teaching Hospital
    b. 37 Military Hospital

SECTION C: TRAINING

7. My training on using the pulse oximeter was adequate.
    a. Strongly disagree
    b. Somewhat disagree
    c. Neither
    d. Somewhat agree
    e. Strongly agree

8. How could pulse oximetry training have been improved? (Select all that apply)
    a. More hands-on or in-person demonstrations
    b. Video demonstrations
    c. Written instructions
    d. Diagrams or figures
    e. Training was sufficient

9. My Training on consenting parents was adequate
    a. Strongly disagree

b. Somewhat disagree
c. Neither
d. Somewhat agree
e. Strongly agree

10. How could your training on consenting parents have been improved? (Select all that apply)
    a. More in-person demonstrations and practice
    b. Video demonstrations
    c. A written script
    d. Diagrams or figures
    e. Training was sufficient

11. I fully understood and was comfortable using the study algorithm.
    a. Strongly disagree
    b. Somewhat disagree
    c. Neither
    d. Somewhat agree
    e. Strongly agree

12. How could understanding of the study algorithm have been improved? (Select all that apply)
    a. More in-person demonstrations and practice
    b. Video demonstrations
    c. Written instructions/explanations
    d. Diagrams/figures
    e. Training was sufficient

13. Was it clear who to report to if you were having issues performing pulse oximetry, using the algorithm or consenting?
    a. Yes
    b. Maybe
    c. No

SECTION D: RECORDS

14. How was the process of obtaining a record of births each morning
    a. Extremely difficult
    b. Somewhat difficult
    c. Neither

d. Somewhat easy
e. Extremely easy

15. How did you locate eligible babies? (Rank methods used)
    a. Written location in. labor and delivery book
    b. Ward notebook records
    c. Electronic records
    d. Going from bed to bed
    e. Asking floor nurses

SECTION E: CONSENT AND PERMISSIONS

16. Did you experience pushback from hospital staff that delayed or interrupted study?
    a. Yes
    b. No

17. If yes, who gave you pushback?
    a. Ward nurses
    b. Labor and delivery nurses
    c. Matrons
    d. Physicians
    e. Other:

18. Reasons for pushback from staff (Select all that apply)
    a. Unaware of study
    b. Interrupting flow of care
    c. Patient about to be discharged
    d. Other:

19. Experiences with parents that prevented or delayed screening (Select all that apply)
    a. Language barriers
    b. Worries about hurting baby
    c. Worries about finding unexpected heart issue
    d. Expected payment/reimbursement
    e. Logistical issues (such as feeding, sleeping, presence of family)
    f. Not interested

20. What would have improved screening success with parents? (Select all that apply)
    a. Prior education before or during pregnancy
    b. Posters/diagrams

c. Assistance form nursing staff
d. Other:
e. Nothing would have helped

SECTION F: WORKING WITH PROVIDERS

21. Did you work with physicians who performed newborn exams during the study?
    a. Yes
    b. No

22. Did you experience challenges working with physicians?
    a. Yes
    b. No

23. If yes to the question above, what challenges did you experience? (Select all that apply)
    a. Lack of clarity on who was scheduled
    b. Physicians not arriving on time
    c. Physicians not arriving at all
    d. Discomfort communicating with physicians
    e. Issues coordinating meeting points
    f. Other:

SECTION G: PULSE OXIMETRY

24. The designated pulse oximeter was always accessible or easy to find.
    a. Strongly disagree
    b. Somewhat disagree
    c. Neither
    d. Somewhat agree
    e. Strongly agree

25. Issues using the pulse oximeter (Select all that apply)
    a. Low battery
    b. Issues with the Velcro foam wrap
    c. Issues with sensor
    d. Oximeter not turning on

26. Average time to obtain stable pulse oximeter reading
    a. <1 minute

b. 1-2 minutes
c. 2-3 minutes
d. 3-4 minutes
e. 5+ minutes

27. Obstacles to obtaining a reading in <1 minute
    a. Crying baby
    b. Restless/fussy baby
    c. Interruption from parents
    d. Interruption from nursing staff
    e. Unable to access right hand or foot

SECTION 8: NEXT STEPS AFTER FAILED BABY IDENTIFIED

28. I was able to follow the study algorithm relatively easily once a failed baby was identified.
    a. Strongly agree
    b. Somewhat disagree
    c. Neither
    d. Somewhat agree
    e. Strongly agree

29. What was most confusing about the study algorithm?
    a. Identifying absolute vs relative fails
    b. Timing of repeat checks
    c. Getting in touch with study team for echocardiography
    d. Nothing was confusing

30. Ways you may have deviated from the protocol:
    a. Repeating pulse oximetry on absolute fails (≤89%)
    b. Not repeating pulse oximetry when there was >3% difference between hand and foot
    c. Screening babies >48 hours old
    d. Other:

31. Was it clear who to contact about a failed baby?
    a. Yes
    b. No

32. Who did you contact for a failed baby?

a. Report in research assistant group chat
b. Call/text Dr. Baafi (research co-investigator)
c. Call/text Prof Yao
d. Notify primary pediatric team

33. Time to contact study team after failed baby identified
    a. Immediately
    b. Within 30 minutes
    c. Within 2 hours
    d. Within 6 hours
    e. Within 12 hours
    f. Within 24 hours

SECTION 9: WORKLOAD

34. I often felt short-staffed.
    a. Strongly disagree
    b. Somewhat disagree
    c. Neither
    d. Somewhat agree
    e. Strongly agree

35. I was often running behind on time.
    a. Somewhat disagree
    b. Somewhat disagree
    c. Neither
    d. Somewhat agree
    e. Strongly agree

Additional comments, suggestions or questions:

____

____

____
